# Appropriate margin for planning target volume for breast radiotherapy during deep inspiration breath-hold by variance component analysis

**DOI:** 10.1186/s13014-021-01777-7

**Published:** 2021-03-06

**Authors:** Yuka Ono, Michio Yoshimura, Tomohiro Ono, Takahiro Fujimoto, Yuki Miyabe, Yukinori Matsuo, Takashi Mizowaki

**Affiliations:** 1 Department of Radiation Oncology and Image-Applied Therapy, Graduate School of Medicine, Kyoto University, 54 Kawahara-cho, Shogoin, Sakyo-ku, Kyoto, Kyoto 606-8507 Japan; 2 Division of Clinical Radiology Service, Kyoto University Hospital, Kyoto, Japan

**Keywords:** Breast cancer, Deep inspiration breath-hold, Cine electronic portal imaging device, Inter-patient, Inter-fraction, Intra-fraction, Planning target volume margin

## Abstract

**Background:**

This study aimed to quantify errors by using a cine electronic portal imaging device (cine EPID) during deep inspiration breath-hold (DIBH) for left-sided breast cancer and to estimate the planning target volume (PTV) by variance component analysis.

**Methods:**

This study included 25 consecutive left-sided breast cancer patients treated with whole-breast irradiation (WBI) using DIBH. Breath-holding was performed while monitoring abdominal anterior–posterior (AP) motion using the Real-time Position Management (RPM) system. Cine EPID was used to evaluate the chest wall displacements in patients. Cine EPID images of the patients (309,609 frames) were analyzed to detect the edges of the chest wall using a Canny filter. The errors that occurred during DIBH included differences between the chest wall position detected by digitally reconstructed radiographs and that of all cine EPID images. The inter-patient, inter-fraction, and intra-fractional standard deviations (SDs) in the DIBH were calculated, and the PTV margin was estimated by variance component analysis.

**Results:**

The median patient age was 55 (35–79) years, and the mean irradiation time was 20.4 ± 1.7 s. The abdominal AP motion was 1.36 ± 0.94 (0.14–5.28) mm. The overall mean of the errors was 0.30 mm (95% confidence interval: − 0.05–0.65). The inter-patient, inter-fraction, and intra-fractional SDs in the DIBH were 0.82 mm, 1.19 mm, and 1.63 mm, respectively, and the PTV margin was calculated as 3.59 mm.

**Conclusions:**

Errors during DIBH for breast radiotherapy were monitored using EPID images and appropriate PTV margins were estimated by variance component analysis.

## Background

Breast-conserving therapy is a standard treatment for early breast cancer to reduce the risk of recurrence and death [1, 2]. However, in the case of left-sided breast cancer, irradiation has been associated with cardiovascular toxicity [3]. Therefore, whole breast irradiation (WBI) with free breathing for left-sided breast cancer patients has increased the incidence of late radiation-induced cardiovascular complications [4–7].

Deep inspiration breath-hold (DIBH) minimizes the dose to the heart and coronary arteries by increasing the distance between the chest wall and the heart [8–12]. However, there have been several reports concerning setup errors during DIBH [13–17]. Hamming et al. reported the variability for surface motion in the left–right (LR), superior-inferior (SI), and anterior–posterior (AP) directions during DIBH for left-sided breast cancer patients by using a surface-guided radiotherapy (SGRT) system [17]. Jensen et al. evaluated chest wall motion during DIBH set only by skin marks and in-room lasers in 65 left-sided breast cancer patients using a cine electronic portal imaging device (cine EPID) [18]. Thus, the appropriate margin for planning target volume (PTV) should take into account the chest wall motion and setup errors during DIBH.

Conventional PTV margin is calculated using the statistical formula proposed by van Herk et al. [19], with errors defined as systematic or individual mean standard deviation (SD) and random or root mean square of individual SDs. Although the formula is widely used in clinical practice, it led to overestimation of systematic errors [20]. Xiao et al. summarized isotropic setup margins for left-sided breast cancer during DIBH using the van Herk formula [16]. These setup margins were large, and so the errors would be overestimated. Recent reports evaluated intra- and inter-fractional chest wall motion during DIBH [19, 21]. However, there are no reports that effectively provide appropriate PTV margins without overestimation of systematic errors. To resolve the overestimation, Matsuo et al. introduced variance component analysis to estimate systematic and random errors [22]. This method analyzed the components derived from patient-related and treatment fraction-related errors as well as residuals for intra- and inter-fractional errors.

Hence, the purpose of this study is to introduce an appropriate PTV margin for left-sided breast cancer radiotherapy during voluntary DIBH (vDIBH) using variance component analysis. Inter- and intra-fractional errors between the chest wall position and reference position during vDIBH derived from the patient-related, fraction-related, and residual errors were detected using cine EPID.

## Methods

### Patient population and computed tomography (CT) simulation

**Fig. 1:**
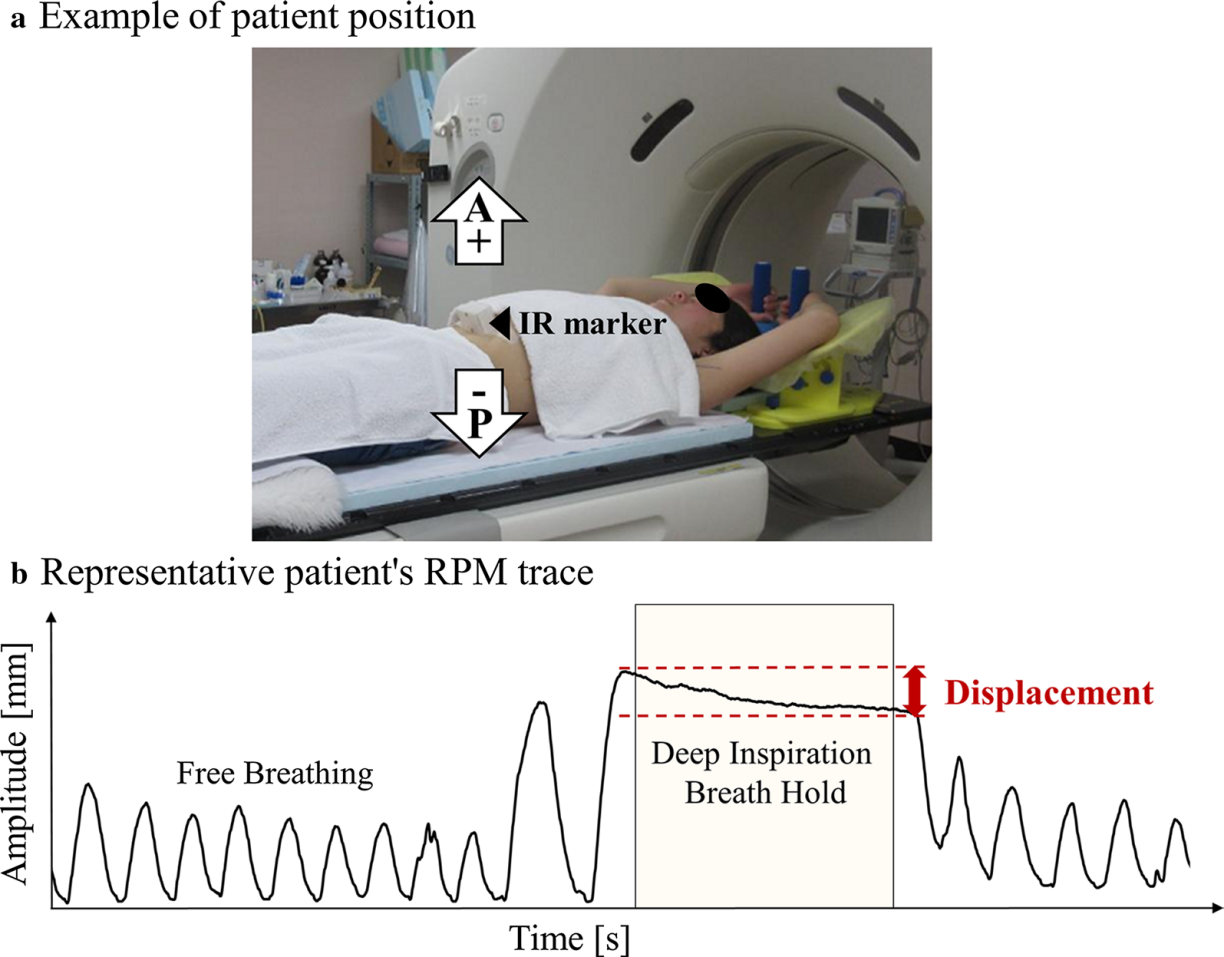
**a** The patient’s position in a breast immobilization device. The infrared marker was placed on the patient’s abdominal wall. **b** Representative RPM trace of the patient. The vertical axis represents the amplitude; the horizontal axis represents time. Dashed lines show the upper and lower gating thresholds with a maximum value. RPM, real-time position management; IR, infrared

All the 25 consecutive left-sided breast cancer patients treated with WBI under vDIBH technique at our institution between January 2017 and June 2018 were enrolled in this study. The median age of the patients was 55 (35–79) years. Patients were immobilized in a supine position, with both arms raised above the head using the Wing Support (Engineering System Co., Ltd., Matsumoto, Nagano, Japan). The infrared (IR) marker was placed on the abdominal wall, between the xiphoid process and the navel, to measure the displacement of the abdominal wall during vDIBH in the AP direction using the Real-time Position Management (RPM) system (Varian Medical Systems, Inc., Palo Alto, CA, USA) without visual feedback (Fig. 1a). While scanning vDIBH-CT images, we confirmed the reproducibility of breath hold by monitoring their abdominal wall motion, and the wall motion was recorded (Fig. 1b). The plain CT images were acquired under free breathing (FB) and vDIBH conditions using SOMATOM Definition AS64 (Siemens, Forchheim, Germany). The CT slice thickness was 2.0 mm.

### Treatment planning

Treatment plans for both FB and vDIBH for WBI were created using the Eclipse planning system v15.6.05 (Varian Medical Systems, Palo Alto, CA, USA) using Acuros XB algorithms. vDIBH was selected for all the patients in this study because of the advantage of dose reduction for organs at risk (OAR), such as the heart, left anterior descending artery (LAD), planning organ at risk volume (PRV) of LAD (PRV-LAD), and the ipsilateral lung. The heart was contoured from where the pulmonary trunk branched into the left and right pulmonary arteries to the apex [23]. The LAD was delineated without intravenous contrast starting from the level the left coronary artery ran into the interventricular groove between the left and right ventricles [24]. The PRV-LAD had 5 mm margins added to the LAD. The breast target and the OARs were delineated according to the ESTRO consensus guideline [25]. The constraints for the OARs adopted were as follows: V_20Gy_ of the ipsilateral lung was less than 15%, the mean dose to the heart was less than 3 Gy. Isocenter was placed at the end of the vertical line from the nipple down to the chest wall on the CT slice containing the center of the nipple. A dose of 42.56 Gy in 16 fractions was given to a reference point located in the CTV based on the International Commission on Radiation Units 62 [26]. The treatment plans were normalized to an isodose prescription line that covered CTV with 90–95% of the prescription dose. All plans consisted of two opposing tangential radiation fields, if necessary with field-in-field, to ensure dose homogeneity within ± 7% in the central axis plane according to the ASTRO guideline [27]. Finally, vDIBH for WBI was selected considering its dosimetric advantages. All the plans were made using TrueBeam (Varian Medical Systems, Palo Alto, CA, USA), with an energy of 6 MV and a dose rate of 600 MU/min.

### Procedure of respiratory gating and irradiation

Before delivering the therapy beam, the therapist checked the match between the light irradiation field and the skin marks during vDIBH; at the same time, the abdominal wall position in the AP direction was monitored by the RPM system. TrueBeam provides an integrated on-board imaging capability for kilovolt and megavolt beams, which offers visual matching with the DRR created using the automatically set planning CT image. As a pre-delivery image guidance, we checked the match of the kV image and DRR image aligned at the sternum. Next, the therapist ensured an agreement of < 3 mm distance between DRR and the acquired MV image, following which beam therapy could be delivered.

During beam delivery, cine EPID images of all fields were taken throughout the entire treatment period and visually checked to confirm stability. EPID was set with a source-to-imager distance (SID) of 150 cm or 160 cm. Meanwhile, RPM was used to monitor the movement of IR markers on the abdominal wall. As shown in Fig. 1a, the amplitude was defined as the difference between the maxima and minima of the abdominal wall position. Radiation oncologists and radiation therapists checked the images and verified the presence of out-of-alignment chest wall position.

### Image processing

To evaluate target motion during beam delivery, chest wall was detected by cine EPID images, retrieved from the ARIA medical imaging database (Varian Medical Systems, Palo Alto, CA, USA) for post-RT analysis. The cine EPID images of the first consecutive 25 patients (309,609 frames) were analyzed. TrueBeam equipped with amorphous silicon-based EPID (aSi-1200) was used. The array has an active detection area of 43 × 43 cm^2^. The image matrix was created from an array of 1280 × 1280 pixels, giving a spatial resolution of 0.5 mm.

**Fig. 2:**
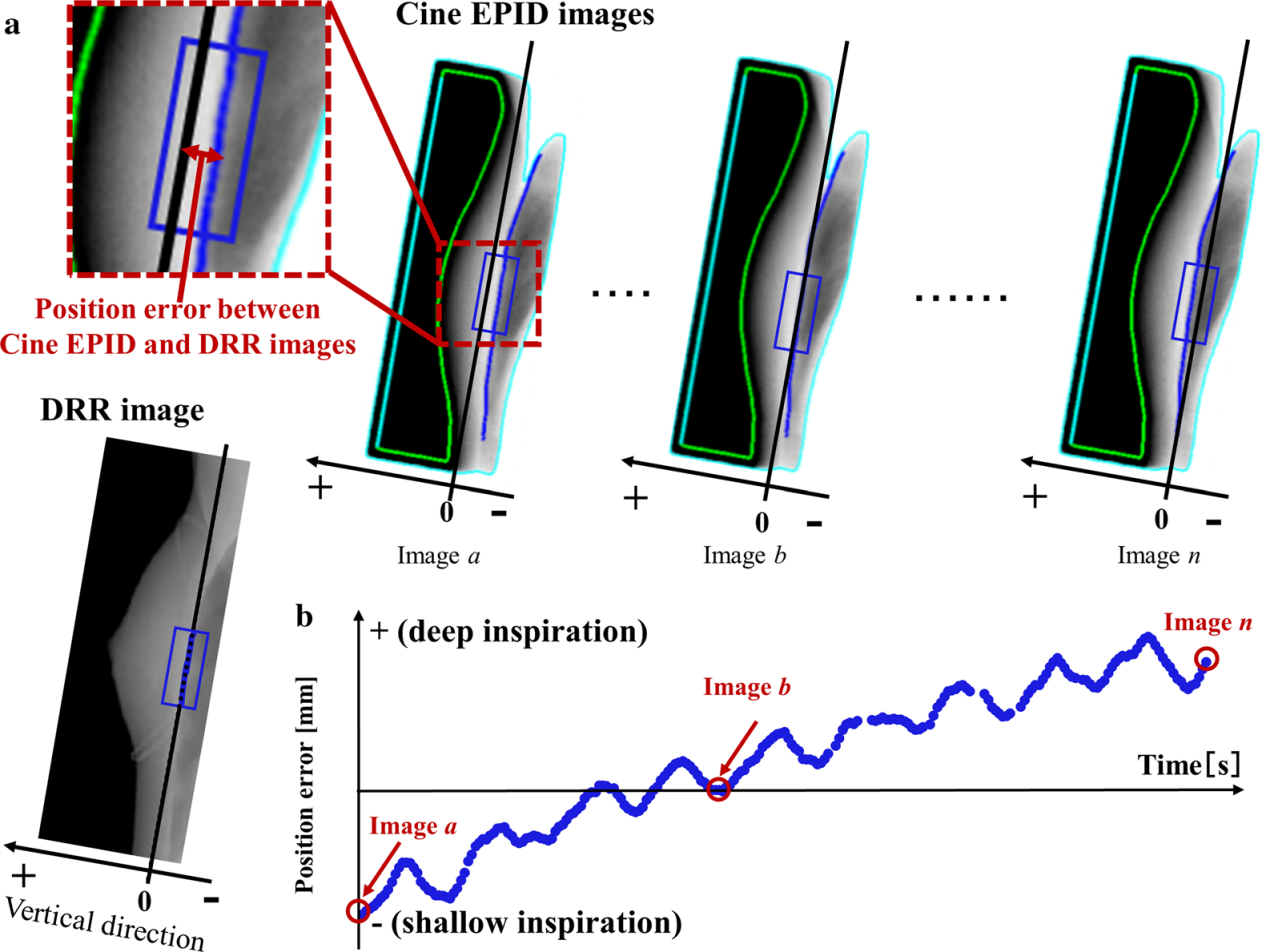
**a** A representative image of the detected edge of chest wall position. The box indicates the ROI used to calculate the position of the chest wall. The chest wall position in the DRR image is set to zero. Images a, b, and n are example images of shallow, just, and deep inspiration, respectively. **b** A representative trace of the position error between cine EPID and DRR images. The vertical axis indicates the position error; the horizontal axis represents time. The points corresponding to images a, b, and n in the above figure are indicated by a circle. ROI, region of interest; DRR, digitally reconstructed radiograph; EPID, electronic portal imaging device

Detection algorithms were programmed using MATLAB (MATLAB 2017b, The MathWorks Inc., Natick, MA, USA) based on a previous detection procedure. A Canny filter detected the edge of the chest wall position in all images. It uses a Gaussian smoothing filter, which removes all artifacts and preserves the chest wall (Fig. 2a). The region of interest of the analysis was set at a level on the isocenter. The positional error of the chest wall between the cine EPID and DRR images were measured along the vertical direction of the long side of the irradiation field in the beams-eye-view plane. The chest wall position in the DRR image was set as the original position. Compared to DRR, the direction of deep inspiration was defined as “+” and that of shallow inspiration as “−” (Fig. 2b).

### Detection accuracy of the in-house software

Before analyzing the patient data, the detection accuracy of the in-house software was evaluated using a breast phantom which had a density and flexibility equivalent to that of soft tissue in the human body [28]. CT images of the breast phantom were acquired, and a treatment plan was created with two opposing tangential fields. Cine EPID images of the breast phantom were acquired without motion during the beam delivery (static condition). Within the ROI, the stability of the chest wall of the phantom was measured along the vertical direction of the long side of irradiation field.

### Evaluation of the errors based on variance component analysis

The errors during vDIBH were defined as differences between the real-time chest wall position on all cine EPID images and the position detected by DRR image in the vertical direction of the irradiation field. Overall mean of the errors during vDIBH, SDs in the error components, and their confidence intervals (CIs) were calculated using the restricted maximum likelihood (REML) method [22].

The conventional definitions for systematic and random errors in the setup of radiotherapy are the SD of individual patient means and the root mean square of individual SDs, respectively. However, this definition overestimates systematic error. Remeijer et al. suggested that SD of the systematic errors would contain a random error component, which would make the true systematic error smaller [20]. The overestimated errors will lead to large margins, which would result in unnecessary irradiation to the OARs. Thus, we calculated the systematic error by variance component analysis. There are several methods for performing variance component analysis. Generally, analysis of variance (ANOVA), which is used for error analysis, is not suitable for unbalanced data. When the number of data points is not equal, REML method is recommended over ANOVA. In this study, the number of measurements were different in all patients, and thus, it is more appropriate to use the REML method.

**Fig. 3:**
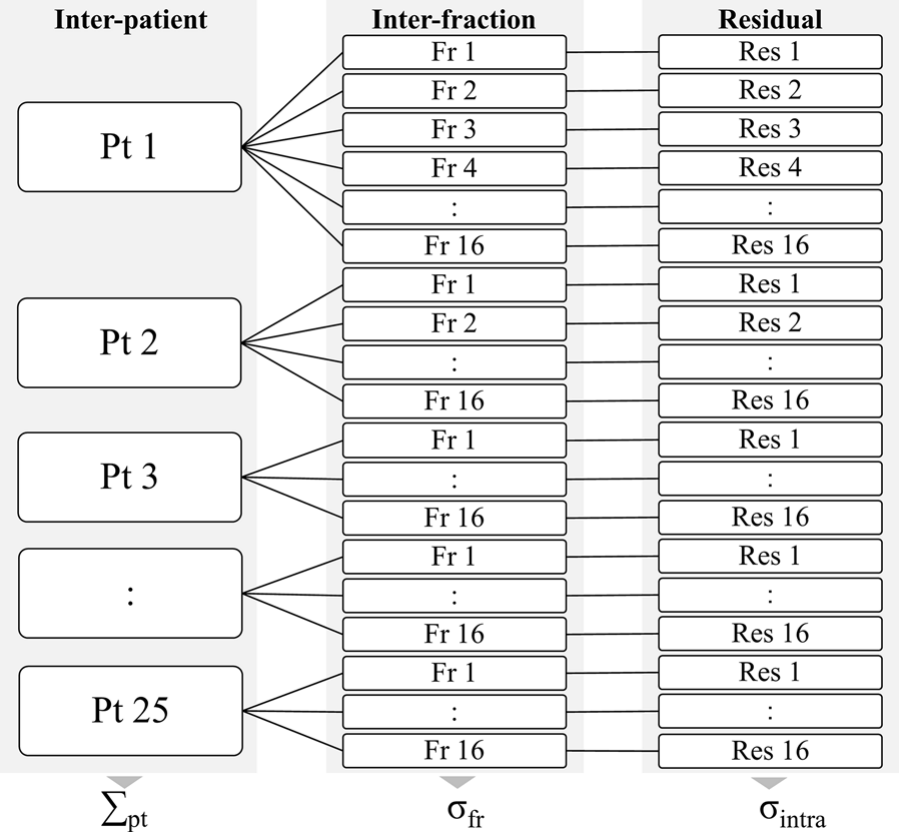
Nested random-effect model of patient, fraction, and residuals. Standard deviations between patients, between fractions, and between residuals were calculated as ∑_pt_, σ_fr_, and σ_intra_, respectively

The errors during vDIBH were evaluated by ∑_pt_, σ_fr_, and σ_intra_, which represent the inter-patient, inter-fraction, and intra-fraction SDs, respectively. Overall mean was defined as the average of all positional errors of the chest wall in all patients. For quantification of components, variance component analysis using REML method was used. A nested random-effect model of the fraction of patients was adopted (Fig. 3), that is, fraction levels are only meaningful within the levels of patient. R version 4.0.0 [29] and lme4 package version 1.1–23 [30] were utilized for statistical analyses.

### Calculation of PTV margin

PTV margin was calculated using the van Herk margin formula [19]:

1 $$2.5\Sigma_{eff} + 0.7\sigma_{eff}$$

where ∑_eff_ and σ_eff_ are the effective values of systematic and random errors, respectively. The coefficients were substituted to ensure that there was 95% minimum dose to the CTV for 90% of patients covered.

∑_eff_ and σ_eff_ are used to examine the systematic effect of the random error under a number of fractions (N) [31]. The ∑_eff_ and σ_eff_ were defined as follows:

2 $$\Sigma_{eff}^{2} = \, \Sigma_{pt}^{2} + \sigma_{fr}^{2} /N$$

3 $$\sigma_{eff}^{2} = \, \left( {1 - 1/N} \right) \, \sigma_{fr}^{2} + \sigma_{{{\text{int}} ra}}^{2}$$

∑_pt_, σ_fr_, and σ_intra_ represent combined values for the inter-patient, inter-fraction, and intra-fraction SDs in the errors during vDIBH, respectively.

## Results

OAR dosimetry with DIBH had many advantages in all the plans. The coverage of the CTV was as follows: D95% was 94.5 (90.5–98.1) and D2% was 106.3 (104.4–110.5). Two sub-fields were used in two patients, to ensure dose conformity. The breath-hold time for all patients was, on average, 20.4 ± 1.7 s (18.1–25.1). For evaluating abdominal wall displacements during irradiation, displacement was defined as the difference in the distance between maxima and minima of the abdominal wall position in the AP direction (1.36 ± 0.94 (0.14–5.28) mm).

Before analyzing the patient error, we evaluated the accuracy of the in-house software. In the static condition of the breast phantom, the displacement of the chest wall in two opposing tangential fields were 0.14 ± 0.07 mm. The pixel size at isocenter plane was 0.47 mm^2^ with an SID of 160 cm. This meant that the detection accuracy of the in-house software was proven to be less than 1 pixel.

The error of the chest wall position for inter-patient (∑_pt_), inter-fraction (σ_fr_), and intra-fraction (σ_intra_) scenarios during vDIBH were 0.82 mm (95% CI: 0.59–1.13), 1.19 mm (95% CI: 1.11–1.29), and 1.63 mm (95% CI: 1.63–1.64), respectively. The overall mean of the REML method was 0.30 mm (95% CI: -0.05–0.65). The numbers of fractions (N) per patient was 16; therefore, after substituting the values into Eqs. (2) and (3), ∑_eff_ and σ_eff_ were calculated as 0.87 mm and 2.00 mm, respectively. Further, using formula (1), the optimal PTV margin for left-sided breast radiotherapy during vDIBH was estimated to be 3.59 mm (Table 1).

## Discussion

This study evaluated the systematic and random errors during vDIBH by the variance component analysis using the REML method. To the best of our knowledge, this is the first report to introduce appropriate PTV margin during vDIBH for radiotherapy of left-sided breast cancer patients derived from the patient, fraction, and residual errors. This approach would contribute to the reduction of the PTV margin, and this would decrease radiation doses to OAR.

The DIBH technique is important for WBI as it reduces radiation dose to the heart and coronary arteries [8, 9, 12] and can be performed cost-effectively [32]. For actual beam delivery, the abdominal wall displacement was 1.36 ± 0.94 mm, monitored by the RPM system. Our study also observed a comparable abdominal wall displacement (2.0 ± 1.0 mm), similar to that seen in Mclntosh’s study [33]. In addition, the overall mean of the chest wall position was 0.30 (95% CI: -0.05–0.65) mm. CIs for the overall mean indicate fundamental error and need to be investigated when the overall mean deviates significantly from zero [34]. In our study, the overall mean did not significantly deviate from zero, indicating a lack of systematic errors in setup procedures; also, the chest wall position was more stable than the abdominal wall position.

The SDs of the chest wall position for inter-patient (∑_pt_), inter-fraction (σ_fr_), and intra-fraction (σ_intra_) scenarios during vDIBH were 0.82, 1.19, and 1.63 mm, respectively (Table 1). The CIs for the systematic and random error of SDs were very small and reliable [34]. These data revealed that all patients could hold their breath steadily, with minor variations. In addition, the systematic and random errors, ∑_eff_ and σ_eff_, were 0.87 mm and 2.00 mm, respectively. Alderliesten et al. investigated the error for DIBH setup using the SGRT system [13] and found systematic and random errors similar to those obtained by Xiao et al. [16]. Our study demonstrated that vDIBH with RPM could be performed stably even without SGRT. In addition, the REML method provided random errors that were comparable to those obtained in other reports [13, 16]. However, it should be noted that the systematic errors seen in other reports were almost the same as the random errors; on the other hand, the systematic errors in our study were smaller than random errors. This is because other studies demonstrated an overestimated systematic error, calculated from the SD of the group mean translational displacements based on the van Herk formula [19]. The wider CI of systematic error would also cause overestimation [22]. In our study, appropriate systematic error was indicated, excluding overestimation.

From the analysis of systematic and random errors, the PTV margin was calculated as 3.59 mm in our study. However, the margins obtained in previous studies were larger than this value owing to the overestimation of systematic errors based on the van Herk formula [16, 35, 36]. Hence, WBI for an overestimated margin for left-sided breast cancer would lead to increase dose to the heart, LAD, lung, and other OAR. Therefore, an appropriate margin should be calculated to deliver the beam to the target with a minimum margin. Here, we revealed the advantage of the application of variance component analysis to PTV margin, and this method can also be applied to other sites. It should be noted that PTV margin was evaluated based on cine EPID images, and the PTV margin meant one direction on BEV plane.

Our technique of appropriate margin calculation might be useful for accelerated partial breast irradiation (APBI) using external beam radiotherapy [23, 37] with or without intensity-modulated radiotherapy (IMRT) [38]. If the internal and setup margins to the lumpectomy cavity were smaller, there would be dosimetric advantages.

Our study has the following limitations. First, only 25 cases were included in this study. In general, the difference between the lower and upper limits of CI tends to be greater as the number of patients and fractions decreases. Similarly, in the present study, the CI for inter-patient SD were larger than those for inter- and intra-fraction SD. Thus, a larger number of patients would be required to narrow the range of the 95% CI of the ∑_pt_ and allow the calculation of a more appropriate margin. Second, only images from the medial and lateral fields can be obtained with this treatment. In EPID image evaluation, chest wall motion represented a combination of LR and AP directions. Additionally, rotational setup error derived from patient positioning was not considered in the current study. Hence, the error in all three dimensions and rotation is unknown. But this can be calculated using SGRT or other systems. For evaluation of breast motion in 3D, Hamming et al. evaluated intra-fractional breath-hold breast motion [17] and reported comparable variability for surface motion in LR, SI, and AP directions. Thus, PTV margin calculated from this study will be useful for application to isotropic margins.

## Conclusions

In this study, the internal and reproducibility margins for DIBH radiotherapy were evaluated by using common positioning devices like Wing Support and a skin mark for position verification. When patients are immobilized (vacuum immobilization) and cone beam CT is performed, there is a possibility of reduction of rotational movement and shrinkage of PTV margin, which is yet another issue.

The inter- and intra-fractional errors derived from patients, fractions, and residuals during vDIBH with RPM were evaluated during chest wall motion using cine EPID. This resulted in appropriate PTV margin calculation, excluding overestimation of systematic errors, thereby reducing unnecessary dose to OAR when compared to conventional methods. The appropriate PTV margins are expected to be applied not only to WBI but also to IMRT and APBI for breast cancer in the near future.

**Table 1:** Detection of errors during vDIBH and requirement for PTV margins

	(mm)	95% CI (mm)
vDIBH error		
Inter-patient (∑_pt_)	0.82	0.59 to 1.13
Inter-fraction (σ_fr_)	1.19	1.11 to 1.29
Intra-fraction (σ_intra_)	1.63	1.63 to 1.64
Overall mean (M)	0.30	− 0.05 to 0.65
Systematic and random error		
Effective systematic (∑_eff_)	0.87	–
Effective random (σ_eff_)	2.00	–
PTV margin	3.59	–

vDIBH, voluntary deep inspiration breath-hold; CI, confidence interval; PTV, planning target volume

## Data Availability

The data that support the findings of this study are available from the corresponding author upon reasonable request.

## Abbreviations

cine EPID: Cine electronic portal imaging device
DIBH: Deep inspiration breath-hold
PTV: Planning target volume
WBI: Whole-breast irradiation
AP: Anterior–posterior
RPM: Real-time Position Management
SDs: Standard deviations
LR: Left–right
SI: Superior-inferior
SGRT: Surface-guided radiotherapy
vDIBH: Voluntary DIBH
IR: Infrared
CTV: Clinical target volume
OAR: Organs at risk
LAD: Left anterior descending artery
PRV: Planning organ at risk volume
MUs: Monitor units
DRR: Digitally reconstructed radiograph
CIs: Confidence intervals
REML: Restricted maximum likelihood
